# Relationship between physical activity and DNA methylation-predicted epigenetic clocks

**DOI:** 10.1038/s41514-025-00217-0

**Published:** 2025-04-12

**Authors:** Yanwei You, Yuquan Chen, Hao Ding, Qiyu Liu, Rui Wang, Kailin Xu, Qingyuan Wang, Danijela Gasevic, Xindong Ma

**Affiliations:** 1 Division of Sports Science & Physical Education, Tsinghua University, Beijing, 100084 China; 2 IDG/McGovern Institute for Brain Research, Tsinghua University, Beijing, 100084 China; 3 School of Public Health and Preventive Medicine, Faculty of Medicine, Nursing & Health Sciences, Monash University, Victoria, 3004 Australia; 4 School of Life Sciences, Tsinghua University, Beijing, 100084 China; 5 Hinda and Arthur Marcus Institute for Aging Research, Hebrew SeniorLife, Harvard Medical School, Boston, MA 02115 USA; 6 The Centre for Global Health, Usher Institute, University of Edinburgh, Edinburgh, EH16 4UX UK

**Keywords:** Biomarkers, Risk factors

## Abstract

This study investigates the relationship between physical activity (PA) levels and DNA methylation (DNAm)-predicted epigenetic clocks in a U.S. population sample (*n* = 948, mean age 62, 49% female). Eight epigenetic clocks were analyzed, revealing that higher PA levels were significantly associated with younger biological ages across all indicators, with the strongest effects observed for SkinBloodAge and LinAge. Multivariable linear regression models, adjusted for sociodemographic and lifestyle factors, highlighted the potential of PA to reduce biological ageing. Subgroup analyses indicated that these associations were more pronounced among non-Hispanic whites, individuals with a BMI of 25–30, and former smokers, suggesting that the impact of PA varies across different groups. These findings emphasize the role of PA in slowing biological ageing and reducing age-related health risks. Promoting regular PA, especially among older adults and those with higher BMI, could improve well-being and lifespan, highlighting PA as a modifiable factor in healthy ageing and age-related disease prevention.

## Introduction

Ageing research increasingly focuses on understanding the biological mechanisms that contribute to ageing and how lifestyle factors, such as physical activity (PA), can influence these processes^1,2^. Among the various indicators of ageing, DNAm-predicted age, including both chronological and phenotypic age (PhenoAge), has gained attention for its ability to provide insights into biological ageing beyond chronological years^3–5^. DNAm patterns, which are epigenetic modifications that occur with age, are influenced by environmental and lifestyle factors, making them crucial indicators for studying ageing^6,7^.

PA is a well-established determinant of health, with evidence showing its protective effects against chronic diseases, cognitive decline, and premature mortality^8–11^. PA has been extensively studied in relation to various biomarkers of ageing, such as telomeres, which are protective caps at the ends of chromosomes that shorten with age^12,13^. Regular PA has been associated with longer telomeres, suggesting a potential protective effect against cellular ageing^14–16^. Additionally, PA has been linked to lower levels of inflammatory markers like C-reactive protein (CRP) and reduced oxidative stress, both of which are closely associated with ageing^17–20^. However, its impact on biological ageing, as measured by DNAm-predicted age, remains an area of active investigation. Specifically, understanding how PA influences various DNAm-based ageing indicators could offer valuable insights into the mechanisms through which PA promotes healthy ageing and longevity.

DNAm-predicted epigenetic clocks include several specific measures such as HorvathAge^21^, HannumAge^22^, SkinBloodAge^23^, LinAge^24^, WeidnerAge^25^, VidalBraloAge^26^, ZhangAge^27^, and PhenoAge^28^. These epigenetic ageing indicators represent different approaches to estimating biological age and have been associated with various health outcomes^29^. While chronological age is simply the number of years lived, DNAm clocks provide a more nuanced view of biological ageing, reflecting physiological functioning and disease risk^30^. Each of these measures captures different aspects of ageing, making them valuable tools for assessing the impact of lifestyle factors like PA.

Recent studies have highlighted the stronger and more consistent associations between PA and epigenetic aging, especially with DNAm GrimAge. For instance, Fox et al.^31^ and Kankaanpää et al.^32^ demonstrated that PA is associated with slower epigenetic aging as measured by GrimAge, with higher PA levels correlating with reduced biological age acceleration. Similarly, Kresovich et al.^33^ and Spartano et al.^34^ found robust associations between PA and biological aging markers across various populations. These findings suggest that PA may influence biological aging processes in a way that is more accurately captured by newer epigenetic clocks^35,36^, such as GrimAge, than by first-generation clocks, which were primarily designed to predict chronological age.

Despite the vast literature on the relationship between PA and biological ageing, existing studies have several limitations. For example, some reviews highlighted the need for more robust studies that investigate the dose-response relationship between PA and various epigenetic ageing markers^31,37,38^. A prospective cohort study conducted in Germany emphasized that while PA has well-documented benefits on ageing and health, its differential impact across socio-demographic groups remains underexplored, particularly in relation to epigenetic ageing^31^. While previous studies primarily focused on chronological age or a single DNAm-predicted clock, such as Horvath’s clock^39^, comprehensive analyses of multiple DNAm-predicted ageing indicators are scarce. Moreover, gaps remain regarding the interaction between lifestyle factors, such as PA, and socio-demographic variables in predicting epigenetic ageing. One study provided an excellent framework for how these subgroup variations should be explored, particularly among different age groups, genders, and ethnicities^40–42^. However, their work does not fully account for how lifestyle factors like PA may modulate these associations across diverse populations.

**Fig. 1:**
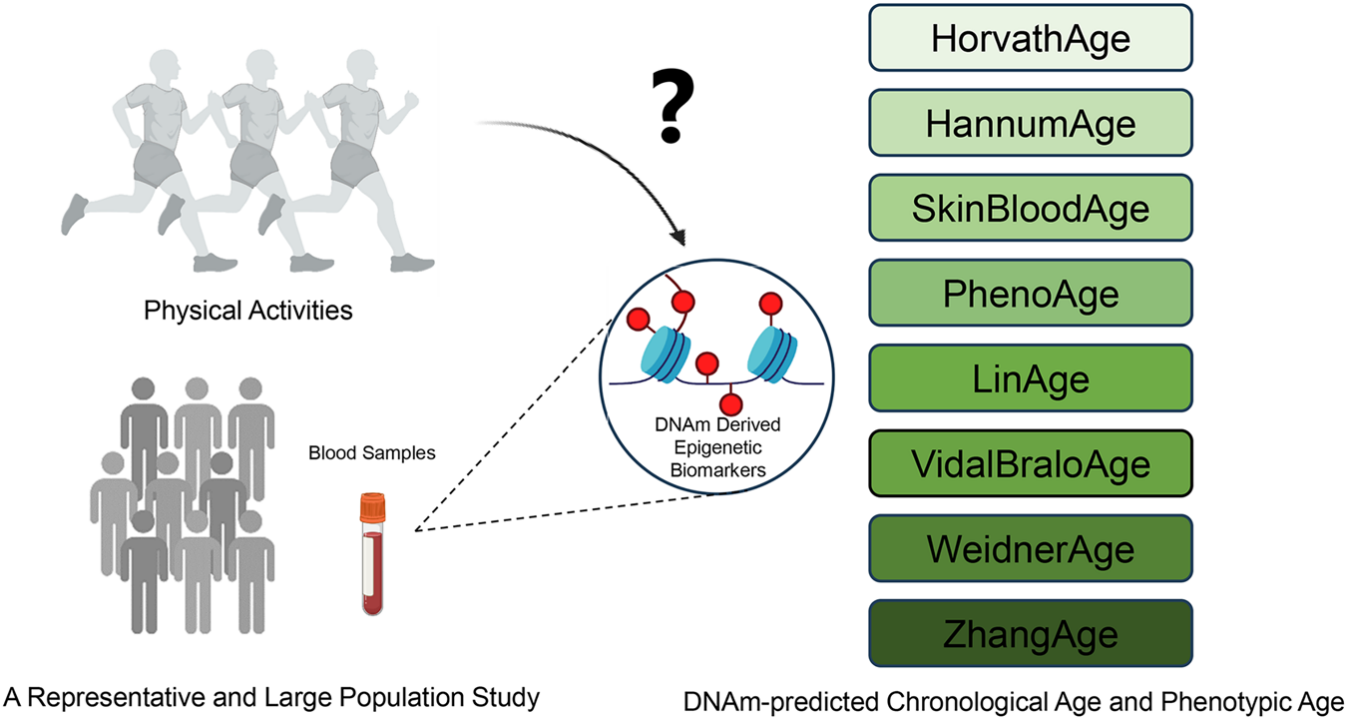
Research Summary and Study Design.

This study aims to address these gaps by examining the relationship between various levels of PA and eight DNAm-predicted epigenetic clocks, while also considering the role of socio-demographic factors. By analyzing a large, nationally representative sample, we aim to provide a more detailed understanding of how PA influences biological ageing and to justify subgroup analyses that can inform tailored public health interventions (Fig. 1). These findings will extend current knowledge by offering a broader perspective on the intersection of PA and epigenetic ageing.

## Results

### Characteristics of study participants

After excluding participants with missing data on exposure, outcome and covariates, the study’s final sample consisted of 948 participants. The mean age is 62.5 years, ranging from 50 to 85, with 50.6% males and 84.3% non-Hispanic White. Participants being active at the lowest level of PA (quartile 1), compared to those being active at a higher level, were more likely to be females, belonging to ethnic minority populations, never married or widowed/divorced, of lower level of formal education, of lower SES (based on poverty-income ratio), and classified as obese (Table 1). In terms of DNAm-predicted ages, participants in the lowest PA quartile had higher LinAge and VidalBraloAge estimates than those in the highest quartiles. Other demographic factors, such as smoking status and alcohol use, showed no notable differences across PA quartiles.

**Table 1:** Demographic characteristics of study participants categorized by physical activity quartiles

Variable	Total participants (*n* = 948)	PA (Q1) (*n* = 235)	PA (Q2) (*n* = 238)	PA (Q3) (*n* = 237)	PA (Q4) (*n* = 238)
Gender					
Male	50.63	45.86	55.25	40.98	60.06
Female	49.37	54.14	44.75	59.02	39.94
Race/ethnicity					
Non-hispanic White	84.31	79.18	81.47	85.92	89.38
Non-hispanic Black	5.56	7.65	6.48	4.89	3.68
Mexican American	2.71	3	3.74	2.1	2.23
Other Race/ethnicity	7.42	10.17	8.31	7.08	4.71
Marital status					
Never married	2.61	3.25	5.26	0.47	1.94
Married/living with partner	74.18	70.49	74.23	73.78	77.63
Widowed/ divorced	23.21	26.26	20.51	25.75	20.43
Education					
Below high school	4.92	7.64	7.34	3.29	2.21
High school	36.67	35.88	35.09	37.89	37.45
College or above	58.42	56.48	57.56	58.82	60.34
Poverty income ratio					
<1	5.79	10.17	7.7	3.56	2.68
[1,3)	30.67	34.05	30.32	33.67	25.28
≥3	63.54	55.78	61.98	62.77	72.03
BMI (kg/m^2^)					
<25	30.18	23.78	25.65	36.06	33.64
[25, 30)	38.84	34.97	38.85	38.87	42.06
≥30	30.98	41.26	35.5	25.08	24.3
Smoking status					
Never smoking	43.28	44.43	45.02	43.55	40.64
Former smoking	45.09	42.62	47.28	42.1	48.22
Current smoking	11.63	12.95	7.7	14.36	11.13
Alcohol use status					
None	32.31	39.21	27.71	37.58	25.27
Moderate alcohol use	60.32	52.26	65.71	55.81	66.97
High alcohol use	7.37	8.53	6.58	6.61	7.76
Age	62.47 ± 0.36	62.51 ± 0.88	63.41 ± 0.72	62.87 ± 0.70	61.29 ± 0.65
HorvathAge	64.35 ± 0.39	64.08 ± 0.73	65.3 ± 0.84	64.17 ± 0.62	63.97 ± 0.54
HannumAge	63.62 ± 0.31	63.55 ± 0.82	64.77 ± 0.65	63.35 ± 0.70	62.99 ± 0.62
SkinBloodAge	61.13 ± 0.36	61.10 ± 0.79	62.27 ± 0.70	61.24 ± 0.69	60.12 ± 0.63
LinAge	54.31 ± 0.50	53.95 ± 1.02	56.43 ± 0.97	54.11 ± 0.82	53.06 ± 0.73
WeidnerAge	52.65 ± 0.48	52.17 ± 0.67	53.21 ± 0.94	53.67 ± 0.88	51.62 ± 0.71
VidalBraloAge	59.53 ± 0.28	58.81 ± 0.53	60.69 ± 0.50	59.83 ± 0.58	58.92 ± 0.53
ZhangAge	65.55 ± 0.13	65.57 ± 0.33	65.97 ± 0.26	65.54 ± 0.25	65.2 ± 0.25
PhenoAge	51.86 ± 0.37	52.02 ± 0.94	53.28 ± 0.76	51.62 ± 0.76	50.81 ± 0.69

*Weighted percentage for category variables and weighted Mean ± SE for continuous variables. *NHANES* National Health and Nutrition Examination Survey, *BMI* body mass index *PA*, physical activity.For PA quartiles, Q1 range from 0 to 1575 Met-min/week; Q2 range from 1575 to 3780 Met-min/week; Q3 range from 3780 to 7897 Met-min/week; Q4 over 7897 Met-min/week.

### Associations of the physical activity with DNA methylation-predicted epigenetic clocks

**Fig. 2:**
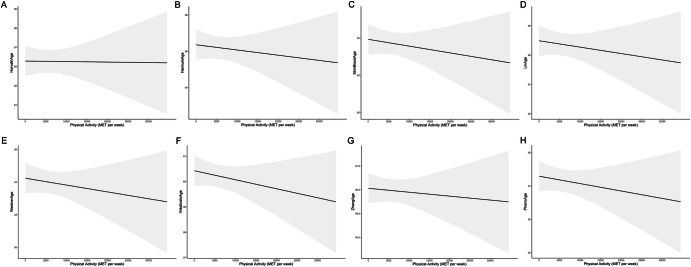
Linear associations between physical activity and DNA methylation-predicted chronological and phenotypic age. **A**–**H** Represent relationships between physical activity and different epigenetic clocks, including **A** HorvathAge, **B** HannumAge, **C** SkinBloodAge, **D** LinAge, **E** WeidnerAge, **F** VidalBraloAge, **G** ZhangAge, and **H** PhenoAge.

In the crude model (Model 1), without any covariate adjustments, higher levels of PA were associated with lower DNAm-predicted ages across all epigenetic clocks (Table 1). After adjusting for gender and race (Model 2), the inverse association between PA and DNAm-predicted ages remained with β-coefficients slightly increasing in magnitude. In the fully adjusted model (Model 3), inverse associations between PA and DNAm-predicted ages were also observed across multiple epigenetic clocks; HorvathAge [β (95% CI): -0.006 (-0.012, -0.001)], HannumAge [β (95% CI): -0.007 (-0.014, -0.001)], SkinBloodAge [β (95% CI): -0.009 (-0.014, -0.004)], LinAge [β (95% CI): -0.012 (-0.018, -0.006)], VidalBraloAge [β (95% CI): -0.005 (-0.010, -0.001)], ZhangAge [β (95% CI): -0.003 (-0.006, -0.001)], and PhenoAge [β (95% CI): -0.007 (-0.014, -0.001)], while the association with WeidnerAge approached statistical significance [β (95% CI): -0.007 (-0.014, 0.000)] (Table 2). Figure 2 further illustrates the linear relationship between PA and DNAm-predicted chronological and phenotypic age using the fully adjusted model. The results indicate that as PA level increases, both DNAm-predicted chronological age and phenotypic age exhibit a significant linear decrease.

**Table 2:** Association between physical activity and DNA methylation-predicted chronological and phenotypic age

	Model 1^a^		Model 2^b^		Model 2^c^	
	β (95% CI)	*p-value*	β (95% CI)	*p-value*	β (95% CI)	*p-value*
HorvathAge	-0.006 (-0.011,-0.002)	0.010	-0.007 (-0.012,-0.002)	0.008	-0.006 (-0.012,-0.001)	0.031
HannumAge	-0.007 (-0.012,-0.002)	0.012	-0.008 (-0.014,-0.003)	0.006	-0.007 (-0.014,-0.001)	0.030
SkinBloodAge	-0.009 (-0.013,-0.005)	<0.001	-0.010 (-0.014,-0.005)	<0.001	-0.009 (-0.014,-0.004)	0.003
LinAge	-0.012 (-0.018,-0.006)	<0.001	-0.013 (-0.019,-0.007)	<0.001	-0.012 (-0.018,-0.006)	0.001
WeidnerAge	-0.009 (-0.016,-0.001)	0.027	-0.009 (-0.017,-0.001)	0.034	-0.007 (-0.014, 0.000)	0.051
VidalBraloAge	-0.005 (-0.009,-0.001)	0.012	-0.006 (-0.010,-0.002)	0.009	-0.005 (-0.010,-0.001)	0.031
ZhangAge	-0.003 (-0.005,-0.001)	0.001	-0.004 (-0.006,-0.002)	0.001	-0.003 (-0.006,-0.001)	0.007
PhenoAge	-0.008 (-0.013,-0.003)	0.003	-0.009 (-0.014,-0.003)	0.002	-0.007 (-0.014,-0.001)	0.026

^a^Model 1, no covariate was adjusted. ^b^Model 2, gender and race were adjusted. ^c^Model 3, gender, race, marital status, education, poverty status, body mass index, smoking status, and alcohol use status were adjusted. CI, confidence interval.

### Subgroup analysis of associations between physical activity and DNA methylation-predicted epigenetic clocks

**Fig. 3:**
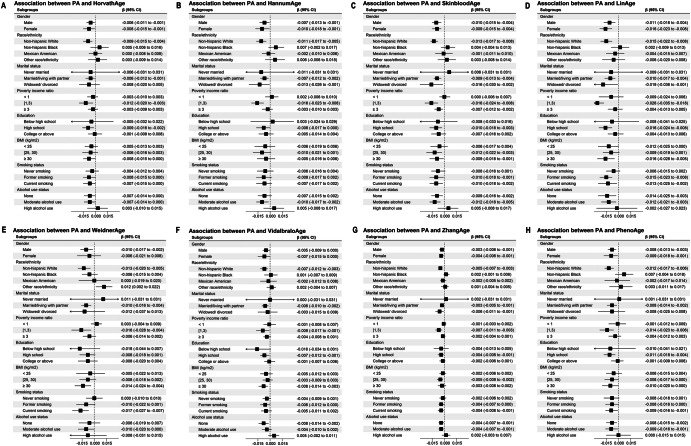
Subgroup analysis of associations between physical activity and DNA methylation-predicted chronological and phenotypic age. **A**–**H** Present stratified results for **A** HorvathAge, **B** HannumAge, **C** SkinBloodAge, **D** LinAge, **E** WeidnerAge, **F** VidalBraloAge, **G** ZhangAge, and **H** PhenoAge.

The subgroup analyses (Fig. 3) consistently show an inverse association between PA and eight DNAm-predicted ages, though the strength of this association varies across demographic and lifestyle factors. For example, in HorvathAge (Table S1), PA is significantly inversely related to predicted age in both males and females, with particularly strong effects in non-Hispanic whites and those with a PIR of 1–3 or a BMI between 25 and 30. Similar patterns were observed for HannumAge (Table S2), where associations with PA were observed for non-Hispanic whites, moderate alcohol consumers, and those with a PIR of 1–3. SkinBloodAge (Table S3) and LinAge (Table S4) follow a similar trend, with significant effects seen across gender, BMI, and smoking status. For WeidnerAge (Table S5), reductions in predicted age are most pronounced in non-Hispanic whites, current smokers, and participants with a PIR of 1–3. VidalBraloAge (Table S6) and ZhangAge (Table S7) also show inverse associations with PA, particularly in males, non-Hispanic whites, and individuals with a BMI of 30 or greater. Finally, for PhenoAge (Table S8), PA is significantly associated with lower predicted ages, especially in non-Hispanic whites, those with a high school education, and current smokers. Full effect sizes and confidence intervals are detailed in the Supplementary Tables.

When examining the interaction terms, we observed that the interaction between PA and demographic or lifestyle factors, such as gender, race/ethnicity, and BMI, was statistically significant in several cases. For example, in HorvathAge (Table S1), the interaction term for gender was not significant (*p* = 0.898), while for race/ethnicity, the interaction term was highly significant (*p* < 0.001), indicating stronger associations between PA and epigenetic age in non-Hispanic whites compared to other racial/ethnic groups. Similar findings were observed in other clocks, such as HannumAge (Table S2) and SkinBloodAge (Table S3), where interaction terms for race/ethnicity were significant, suggesting that the relationship between PA and epigenetic age is modified by racial/ethnic background. These findings highlight the importance of considering interactions in subgroup analyses, which can reveal more nuanced relationships between PA and biological aging across different populations. Full effect sizes and confidence intervals are detailed in Supplementary Tables.

## Discussions

This study explored the association between PA levels and DNAm-predicted epigenetic clocks in a large, nationally representative sample of the U.S. population. The findings support an inverse relationship between PA and biological aging, as indicated by several epigenetic ageing indicators, including HorvathAge, HannumAge, SkinBloodAge, and PhenoAge. Unlike prior research that focused on a limited number of aging biomarkers, this study examines the impact of PA across eight different epigenetic clocks. Additionally, subgroup analyses based on BMI, race, and other socio-demographic factors provide insights into how PA may differentially affect biological aging across populations, highlighting its potential as an intervention for promoting healthy aging.

Our results suggest that higher levels of PA are significantly associated with younger biological ages across multiple epigenetic clocks, even after adjusting for confounding factors such as gender, race, BMI, smoking status, and alcohol consumption. Specifically, SkinBloodAge and LinAge showed the strongest inverse associations, implying that PA exerts a profound impact on epigenetic mechanisms related to biological ageing. However, there is also strength difference of associations between PA and different DNAm Age indicators. This discrepancy could be due to several factors, such as specific measures of biological aging used. In our study, our analysis focused on various subgroups, which could reveal associations that may have been masked in previous studies using more homogeneous samples or different analytical approaches. These findings align with previous studies linking regular PA to beneficial health outcomes, such as reduced inflammation^43–46^, lower oxidative stress^47,48^, and enhanced metabolic function^49,50^, which are key contributors to slower biological ageing^51–54^.

The observed interactions between physical activity (PA) and biological aging in different subgroups, particularly in current smokers, warrant further discussion. Our finding that PA is more strongly associated with lower epigenetic age in current smokers may indeed be influenced by residual confounding, particularly related to smoking behavior. Smoking status, as assessed in this study, is a crude measure that does not account for the intensity or duration of smoking. It is possible that physically active individuals tend to smoke less than their less active counterparts, which could explain the stronger associations observed in smokers. This potential residual confounding is highlighted by a previous literature^32^, which found that adjusting for smoking status attenuated the associations between physical activity and epigenetic aging. In their study, smoking was shown to be a significant confounder in the relationship between lifestyle factors and epigenetic age acceleration, suggesting that smoking may reflect a broader pattern of unhealthy behaviors that also affect biological aging. While our study adjusted for smoking status as a covariate, we acknowledge that further refinements, such as accounting for smoking intensity or cumulative smoking exposure, may be necessary to more accurately isolate the effects of PA on biological aging.

The observation that participants in the highest PA quartiles had lower LinAge and VidalBraloAge further emphasizes the dose-dependent effect of exercise on slowing biological ageing. Additionally, our findings offer new evidence on the protective effects of PA against epigenetic ageing in individuals with lower BMIs (below 30 kg/m^2^), underscoring the importance of weight management in enhancing the anti-aging benefits of exercise. This suggests that maintaining a healthy weight may enhance the protective effects of PA on ageing, which was consistent with previous literature^34,55,56^. Moreover, a strong negative association observed in non-Hispanic whites indicates potential genetic or lifestyle factors that could modify the relationship between PA and epigenetic ageing markers. These findings highlight the role of lifestyle factors in modulating the impact of exercise on biological ageing^57–59^. To sum up, our exploration underscores the need for further research to investigate how different demographic groups may benefit differently from PA interventions^31,34^.

The findings from this study have important public health implications. With ageing populations worldwide, promoting regular PA could be a key strategy for delaying the onset of age-related diseases and enhancing longevity^60^. The robust inverse associations between PA and epigenetic ageing markers suggest that even modest increases in PA could yield significant benefits in terms of biological age reduction. Public health initiatives aimed at encouraging PA, particularly among older adults and individuals with higher BMIs, could play a crucial role in improving population health and reducing healthcare burdens associated with ageing^60–62^. Tsinghua University has a tradition of “no sports, no Tsinghua”. The first director of the Tsinghua Physical Education Department, Mr. John Mo, once said, “Sports have transferable value.” In the midst of busy studies and work, physical activity can improve energy levels, enhance sleep quality, and strengthen the body’s recovery abilities, enabling individuals to face various challenges in life more effectively. Therefore, incorporating physical activities into daily life can not only delay aging but also help maintain optimal performance throughout a long career.

In our study, we primarily focused on first-generation epigenetic clocks, such as Horvath’s and Hannum’s clocks, due to their availability in the NHANES dataset. While these clocks have been widely used in aging research, recent studies have shown that newer epigenetic clocks^63^, such as DNAm GrimAge^64^ and DunedinPACE^65^, provide more accurate predictions of biological aging and are more strongly associated with health outcomes, including chronic conditions and mortality^66^. Our decision to exclude these newer estimators from our analysis was primarily due to the limitations of the available data, as the necessary variables for calculating these clocks were not included in the NHANES dataset.

While this study provides valuable insights into the relationship between PA and biological ageing, there are several limitations to consider. First, the cross-sectional design precludes us from establishing causality between PA and DNAm-predicted age. Longitudinal studies are needed to confirm these associations and explore the long-term effects of PA on biological ageing. Second, the study relied on self-reported PA data, which may be subject to recall bias and inaccuracies. Objective measures of PA, such as accelerometry, could provide more precise estimates of activity levels in future research. Additionally, the observed associations in this study may be influenced by the fact that chronologically older individuals tend to exercise less, which could confound the interpretation of the results. Due to the restricted access to certain DNAm data, we were unable to calculate age acceleration measures as outcomes, which could have provided more direct insight into the relationship between physical activity and biological aging. Future studies should aim to explore the mechanisms underlying the relationship between PA and epigenetic ageing, including the role of inflammation, oxidative stress, and metabolic pathways.

In conclusion, this study provides evidence that higher levels of PA are significantly associated with lower DNAm-predicted chronological and phenotypic ages, indicating that regular PA may play a crucial role in slowing biological ageing. These findings contribute to the growing body of literature on the benefits of PA, particularly its potential to mitigate the effects of ageing at the molecular level. The consistent inverse relationships observed across multiple epigenetic clocks, even after adjusting for confounding factors, suggest that promoting PA could serve as an effective public health strategy for enhancing healthy ageing and reducing age-related disease risks.

## Methods

### Study population

The National Health and Nutrition Examination Survey (NHANES, administered by the National Center for Health Statistics (NCHS) under the Centers for Disease Control and Prevention (CDC) in the United States, is a comprehensive, nationwide survey designed to evaluate the health and nutritional status of the non-institutionalized U.S. population. The survey employs a complex, stratified, multistage probability sampling method and collects data through structured household interviews, visits to mobile examination centers, and laboratory tests. The study protocol received approval from the NCHS Ethics Review Board, with all participants providing written informed consent.

**Fig. 4:**
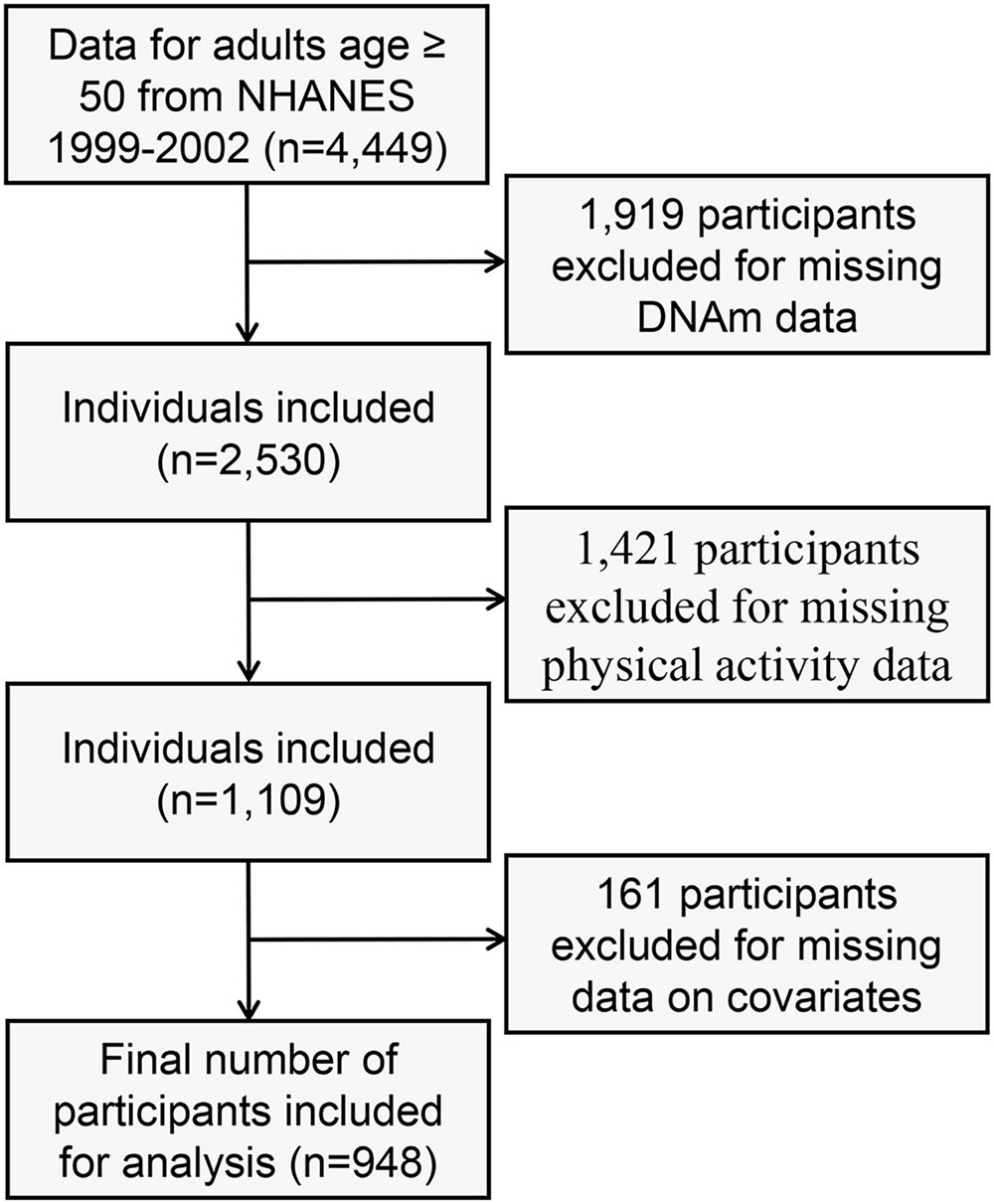
Flow chart of the study design.

This study specifically focused on the DNAm data available from the 1999–2000 and 2001–2002 NHANES cycles, which included participants aged 50 years and older who had provided blood samples for DNA analysis. The initial sample consisted of 4449 individuals, representing a random selection of about half of the eligible non-Hispanic White participants, along with all eligible non-Hispanic Black, Mexican American, other Hispanic, and other race participants. After excluding individuals with missing DNAm data (*n* = 1919), missing PA data (*n* = 1421), or missing data on covariates (*n* = 161), the final analysis included 948 participants (Fig. 4).

### Exposure variable: Physical Activity

PA levels were assessed based on participants’ self-reported involvement in various activities, such as walking, swimming, and playing ball games, over the previous month. The Physical Activity Questionnaire (PAQ) section of NHANES, which includes a comprehensive set of questions about daily routines, leisure activities, and sedentary behaviors, was the primary tool used for data collection. This questionnaire includes several items that had been featured in earlier NHANES cycles or other federal surveys^67^.

Participants were asked to document the number of days in the past month they engaged in each specific activity and to estimate the typical duration of each session in minutes. NHANES provided definitions for classifying activities as either moderate or vigorous, and each activity was assigned a specific metabolic equivalent of task (MET) value corresponding to its intensity^68^. The MET values, drawn from the compendium of physical activities, were assigned to 62 different activities as listed on the PAQ part of the official NHANES website^69^.

Referring to previous NHANES studies^70–72^, the overall PA level of each individual was quantified by summing the total minutes of activities reported by participants. The MET values were multiplied by the time and frequency of each reported activity to calculate MET-minutes per week, which reflects the ratio of a participant’s energy expenditure during PA compared to resting metabolic rate. This comprehensive measurement allows for an estimation of the total MET/minutes per week, providing a detailed assessment of each participant’s level of PA^73^.

### Outcome variable: DNAm predicted epigenetic clocks

DNA of eligible participants was extracted from whole blood samples and stored at -80 °C. The DNAm assay was conducted at Dr. Yongmei Liu’s laboratory at Duke University. DNA samples (500 ng) were bisulfite converted using the Zymo EZ DNAm kit, following the manufacturer’s instructions. The bisulfite-treated DNA was then analyzed using the Illumina Infinium MethylationEPIC BeadChip array, generating methylation data across approximately 850,000 CpG sites. The initial data output consisted of IDAT files, containing fluorescence intensities from the red and green channels of the BeadChip array. These signals were transformed into methylated and unmethylated intensities. Background subtraction was performed using control probes, and color correction was applied to account for variations between the two channels.

Outlier samples were identified and excluded if the median intensity values for both methylated and unmethylated channels were below a threshold of 10.5. Imputation of missing data was performed using the DNAm epigenetic biomarker creators’ methods^28,39^. For certain biomarkers, a gold standard reference dataset was employed, while for others, the NHANES dataset itself was used. To correct for probe design bias due to differences in CpG density, the Beta Mixture Quantile (BMIQ) normalization method was employed. This method adjusted type 2 probes against a gold standard reference or type 1 probes, depending on the biomarker being analyzed. Details of samples processing can be found at NHANES website^74^.

Further quality control steps included functional normalization and detection of outlier samples and probes. Probes with a performance *p*-value greater than 1 × 10^-16, SNP-containing probes, polymorphic probes, and cross-hybridizing probes were removed. Technical variation due to factors such as plate, chip, and row number was corrected using the ComBat method. This methodology ensured the robustness and reliability of the DNAm data, making it suitable for downstream analyses in various health-related studies

DNAm-based epigenetic biomarkers were produced using coefficients provided by the creators of each biomarker [HorvathAge^21^, HannumAge^22^, SkinBloodAge^23^, LinAge^24^, WeidnerAge^25^, VidalBraloAge^26^, ZhangAge^27^, and PhenoAge^28^]. The resulting biomarkers included in this study are estimates for chronological age and phenotypic age.

### Covariates

The selection of covariates was based on the available evidence^75–78^. This study took into account several covariates, including gender, race/ethnicity (categorized as Non-Hispanic White, Non-Hispanic Black, Mexican American, and Other Race/Ethnicity), education level (classified as Below High School, High School, and College or Above), and marital status (Never Married, Married/Living with Partner, Widowed/Divorced). Additional factors included the poverty-to-income ratio (<1, ≥1 and <3, ≥3), Body mass index (<25, ≥25 and <30, ≥30.0), smoking status (Never Smoked, Former Smoker, Current Smoker), and alcohol consumption (None, Moderate, High). Detailed descriptions of the measurement procedures for these variables are available on the CDC NHANES website at https://www.cdc.gov/nchs/nhanes/.

### Statistical analysis

Statistical analyses were performed in line with CDC guidelines, accessible at https://www.cdc.gov/nchs/nhanes/tutorials/default.aspx. Participants characteristics were presented by PA level quartiles. Continuous variables were reported as weighted means with standard errors (Mean ± SE), while categorical variables were presented as weighted percentages.

Special sample weights are required to analyze the DNAm data properly. Specific sample weights for this subsample, WTDN4YR, are included in these data files and should be used when analyzing these data. The sample weights created for this file used the examination sample weight, i.e., WTMEC4YR, as the base weight. This base weight was adjusted for additional nonresponse to these array tests and re-poststratified to the population total using gender, age, and race/Hispanic origin. It is important to note that participants who were part of the eligible population but did not provide a blood specimen for DNA, did not have sufficient volume of DNA specimens, or did not give consent for their specimens to be used for future research are included in the data file; however, they have a sample weight assigned as “0” in their records.

Three weighted regression models were constructed to assess the relationship between PA and DNAm-based chronological and phenotypic ages. The first model (Crude model 1) included no covariate adjustments. Model 2 accounted for gender and race, and Model 3 incorporated adjustments for gender, race, marital status, education level, poverty status, body mass index, smoking habits, and alcohol consumption. To further investigate the association between PA and DNAm-predicted chronological and phenotypic ages, a multivariable linear regression model was employed based on the fully adjusted Model 3, allowing for visualization of the relationships between these variables. Additionally, subgroup analyses and interaction tests were carried out to explore these relationships across different subgroups.

All analyses were conducted using R version 4.3.3, with a two-tailed p-value of less than 0.05 deemed statistically significant.

## Supplementary information

Supplementary Materials

## Data Availability

No datasets were generated or analyzed during the current study.
