# Supplementary material for: Relationship between physical activity and DNA methylation-predicted epigenetic clocks
Source: NPJ Aging. 2025 Apr 12;11(1):27. doi: 10.1038/s41514-025-00217-0 (PMC11993638; doi:10.1038/s41514-025-00217-0)
Supplement: Supplementary file 1 — Supplementary Materials [file 41514_2025_217_MOESM1_ESM.pdf]

**Abbreviations List:**

PA: Physical Activity
DNAm: DNA Methylation
BMI: Body Mass Index
PIR: Poverty Income Ratio
HorvathAge: Horvath's Epigenetic Age
HannumAge: Hannum's Epigenetic Age
SkinBloodAge: Skin and Blood Epigenetic Age
LinAge: Lin’s Epigenetic Age
WeidnerAge: Weidner’s Epigenetic Age
VidalBraloAge: Vidal-Bralo Epigenetic Age
ZhangAge: Zhang’s Epigenetic Age
PhenoAge: Phenotypic Age

For HorvathAge (Table S1), PA was significantly inversely associated in both males (β = -0.01, 95% CI: -0.015 to -0.004) and females (β = -0.009, 95% CI: -0.015 to -0.004). This association was particularly strong in non-Hispanic whites (β = -0.013, 95% CI: -0.017 to -0.008) and participants with a poverty income ratio (PIR) of 1–3 (β = -0.016, 95% CI: -0.024 to -0.008), as well as those with a BMI between 25 and 30 (β = -0.012, 95% CI: -0.022 to -0.003) and former smokers (β = -0.01, 95% CI: -0.018 to -0.001).

Table S1. Subgroup analysis of the association between physical activity and DNA methylation-predicted HorvathAge

| Subgroups | β (95% CI) | p-value | p for interaction |
|---|---|---|---|
| Gender | | | 0.898 |
| Male | -0.01(-0.015,-0.004) | <0.001 | |
| Female | -0.009(-0.015,-0.004) | <0.001 | |
| Race/ethnicity | | | < 0.001 |
| Non-hispanic White | -0.013(-0.017,-0.008) | <0.0001 | |
| Non-hispanic Black | 0.004(-0.004,0.013) | 0.298 | |
| Mexican American | -0.001(-0.011,0.010) | 0.902 | |
| Other race/ethnicity | 0.003(-0.008,0.014) | 0.446 | |
| Marital status | | | 0.059 |
| Never married | 0.006(-Inf,Inf) | 0.296 | |
| Married/living with partner | -0.009(-0.013,-0.004) | <0.001 | |
| Widowed/ divorced | -0.016(-0.030,-0.002) | 0.029 | |
| Poverty income ratio | | | 0.021 |
| < 1 | 0(-0.006,0.007) | 0.923 | |
| [1,3) | -0.016(-0.024,-0.008) | <0.001 | |
| ≥ 3 | -0.007(-0.012,-0.002) | 0.012 | |
| Education | | | 0.798 |
| Below high school | -0.008(-0.033,0.016) | 0.491 | |
| High school | -0.01(-0.018,-0.003) | 0.006 | |
| College or above | -0.007(-0.016,0.002) | 0.107 | |
| BMI (kg/m$^2$) | | | 0.676 |
| < 25 | -0.006(-0.017,0.004) | 0.209 | |
| [25, 30) | -0.012(-0.022,-0.003) | 0.010 | |
| ≥ 30 | -0.009(-0.018,-0.001) | 0.029 | |
| Smoking status | | | 0.916 |
| Never smoking | -0.008(-0.016,0.001) | 0.079 | |
| Former smoking | -0.01(-0.018,-0.001) | 0.032 | |
| Current smoking | -0.01(-0.018,-0.002) | 0.021 | |
| Alcohol use status | | | 0.075 |
| None | -0.009(-0.016,-0.002) | 0.010 | |
| Moderate alcohol use | -0.012(-0.018,-0.005) | 0.001 | |
| High alcohol use | 0.005(-0.008,0.017) | 0.447 | |

Similarly, for HannumAge (Table S2), PA was associated with lower age predictions in both males (β = -0.007, 95% CI: -0.013 to -0.001) and females (β = -0.01, 95% CI: -0.018 to -0.001). Non-Hispanic whites (β = -0.011, 95% CI: -0.017 to -0.005), participants with a PIR of 1–3 (β = -0.016, 95% CI: -0.023 to -0.008), and moderate alcohol consumers (β = -0.01, 95% CI: -0.017 to -0.002) demonstrated particularly strong negative associations with HannumAge.

Table S2. Subgroup analysis of the association between physical activity and DNA methylation-predicted HannumAge.

| Subgroups | β (95% CI) | p-value | p for interaction |
|---|---|---|---|
| Gender | | | 0.452 |
| Male | -0.007(-0.013,-0.001) | 0.023 | |
| Female | -0.01(-0.018,-0.001) | 0.023 | |
| Race/ethnicity | | | < 0.0001 |
| Non-hispanic White | -0.011(-0.017,-0.005) | <0.001 | |
| Non-hispanic Black | 0.007(-0.002,0.017) | 0.116 | |
| Mexican American | -0.002(-0.010,0.006) | 0.587 | |
| Other race/ethnicity | 0.006(-0.006,0.018) | 0.211 | |
| Marital status | | | 0.015 |
| Never married | 0.011(-Inf,Inf) | 0.176 | |
| Married/living with partner | -0.007(-0.012,-0.002) | 0.009 | |
| Widowed/ divorced | -0.013(-0.026,-0.001) | 0.042 | |
| Poverty income ratio | | | 0.003 |
| < 1 | 0.002(-0.006,0.010) | 0.577 | |
| [1,3) | -0.016(-0.023,-0.008) | <0.001 | |
| ≥ 3 | -0.003(-0.010,0.003) | 0.289 | |
| Education | | | 0.675 |
| Below high school | 0.003(-0.024,0.029) | 0.816 | |
| High school | -0.008(-0.017,0.000) | 0.049 | |
| College or above | -0.005(-0.014,0.004) | 0.280 | |
| BMI (kg/m$^2$) | | | 0.834 |
| < 25 | -0.006(-0.019,0.006) | 0.326 | |
| [25, 30) | -0.01(-0.021,0.001) | 0.065 | |
| ≥ 30 | -0.005(-0.016,0.006) | 0.333 | |
| Smoking status | | | 0.962 |
| Never smoking | -0.006(-0.016,0.004) | 0.229 | |
| Former smoking | -0.008(-0.017,0.002) | 0.126 | |
| Current smoking | -0.007(-0.017,0.002) | 0.106 | |
| Alcohol use status | | | 0.182 |
| None | -0.007(-0.015,0.002) | 0.115 | |
| Moderate alcohol use | -0.01(-0.017,-0.002) | 0.013 | |
| High alcohol use | 0.005(-0.008,0.017) | 0.434 | |

In the case of SkinBloodAge (Table S3), PA was significantly associated with lower predicted ages in both males (β = -0.01, 95% CI: -0.015 to -0.004) and females (β = -0.009, 95% CI: -0.015 to -0.004). The effect was most pronounced in non-Hispanic whites (β = -0.013, 95% CI: -0.017 to -0.008) and participants with a PIR of 1–3 (β = -0.016, 95% CI: -0.024 to -0.008). Notably, those with a BMI between 25 and 30 also showed a significant reduction in SkinBloodAge (β = -0.012, 95% CI: -0.022 to -0.003).

Table S3. Subgroup analysis of the association between physical activity and DNA methylation-predicted SkinBloodAge.

| Subgroups | β (95% CI) | p-value | p for interaction |
|---|---|---|---|
| Gender | | | 0.898 |
| Male | -0.01(-0.015,-0.004) | <0.001 | |
| Female | -0.009(-0.015,-0.004) | <0.001 | |
| Race/ethnicity | | | < 0.0001 |
| Non-hispanic White | -0.013(-0.017,-0.008) | <0.0001 | |
| Non-hispanic Black | 0.004(-0.004,0.013) | 0.298 | |
| Mexican American | -0.001(-0.011,0.010) | 0.902 | |
| Other race/ethnicity | 0.003(-0.008,0.014) | 0.446 | |
| Marital status | | | 0.059 |
| Never married | 0.006(-Inf,Inf) | 0.296 | |
| Married/living with partner | -0.009(-0.013,-0.004) | <0.001 | |
| Widowed/ divorced | -0.016(-0.030,-0.002) | 0.029 | |
| Poverty income ratio | | | 0.021 |
| < 1 | 0(-0.006,0.007) | 0.923 | |
| [1,3) | -0.016(-0.024,-0.008) | <0.001 | |
| ≥ 3 | -0.007(-0.012,-0.002) | 0.012 | |
| Education | | | 0.798 |
| Below high school | -0.008(-0.033,0.016) | 0.491 | |
| High school | -0.01(-0.018,-0.003) | 0.006 | |
| College or above | -0.007(-0.016,0.002) | 0.107 | |
| BMI (kg/m$^2$) | | | 0.676 |
| < 25 | -0.006(-0.017,0.004) | 0.209 | |
| [25, 30) | -0.012(-0.022,-0.003) | 0.010 | |
| ≥ 30 | -0.009(-0.018,-0.001) | 0.029 | |
| Smoking status | | | 0.916 |
| Never smoking | -0.008(-0.016,0.001) | 0.079 | |
| Former smoking | -0.01(-0.018,-0.001) | 0.032 | |
| Current smoking | -0.01(-0.018,-0.002) | 0.021 | |
| Alcohol use status | | | 0.075 |
| None | -0.009(-0.016,-0.002) | 0.010 | |
| Moderate alcohol use | -0.012(-0.018,-0.005) | 0.001 | |
| High alcohol use | 0.005(-0.008,0.017) | 0.447 | |

For LinAge (Table S4), PA was negatively associated with predicted age in both males (β = -0.011, 95% CI: -0.018 to -0.004, p = 0.005) and females (β = -0.016, 95% CI: -0.023 to -0.008, p < 0.001). Non-Hispanic whites (β = -0.015, 95% CI: -0.022 to -0.008) and those with a BMI of 30 or greater (β = -0.016, 95% CI: -0.026 to -0.005) exhibited significant reductions in LinAge.

Table S4. Subgroup analysis of the association between physical activity and DNA methylation-predicted LinAge.

| Subgroups | β (95% CI) | p-value | p for interaction |
|---|---|---|---|
| Gender | | | 0.312 |
| Male | -0.011(-0.018,-0.004) | 0.005 | |
| Female | -0.016(-0.023,-0.008) | <0.001 | |
| Race/ethnicity | | | 0.023 |
| Non-hispanic White | -0.015(-0.022,-0.008) | <0.001 | |
| Non-hispanic Black | 0.002(-0.009,0.013) | 0.712 | |
| Mexican American | -0.004(-0.015,0.007) | 0.434 | |
| Other race/ethnicity | -0.006(-0.020,0.008) | 0.320 | |
| Marital status | | | 0.387 |
| Never married | -0.009(-Inf,Inf) | 0.256 | |
| Married/living with partner | -0.01(-0.017,-0.004) | 0.004 | |
| Widowed/ divorced | -0.019(-0.036,-0.001) | 0.037 | |
| Poverty income ratio | | | 0.002 |
| < 1 | -0.009(-0.024,0.006) | 0.200 | |
| [1,3) | -0.026(-0.035,-0.018) | <0.0001 | |
| ≥ 3 | -0.004(-0.013,0.005) | 0.407 | |
| Education | | | 0.127 |
| Below high school | -0.008(-0.041,0.025) | 0.609 | |
| High school | -0.016(-0.024,-0.008) | <0.001 | |
| College or above | -0.004(-0.013,0.006) | 0.403 | |
| BMI (kg/m$^2$) | | | 0.724 |
| < 25 | -0.012(-0.025,0.000) | 0.043 | |
| [25, 30) | -0.009(-0.019,0.001) | 0.063 | |
| ≥ 30 | -0.016(-0.026,-0.005) | 0.006 | |
| Smoking status | | | 0.237 |
| Never smoking | -0.006(-0.015,0.002) | 0.147 | |
| Former smoking | -0.015(-0.023,-0.008) | <0.001 | |
| Current smoking | -0.013(-0.025,-0.002) | 0.023 | |
| Alcohol use status | | | 0.612 |
| None | -0.014(-0.025,-0.003) | 0.017 | |
| Moderate alcohol use | -0.012(-0.021,-0.003) | 0.009 | |
| High alcohol use | -0.002(-0.027,0.023) | 0.855 | |

For WeidnerAge (Table S5), a similar trend was observed, with significant inverse associations between PA and age predictions in males (β = -0.01, 95% CI: -0.017 to -0.002) and non-Hispanic whites (β = -0.013, 95% CI: -0.020 to -0.005). Additionally, participants with a PIR of 1–3 (β = -0.016, 95% CI: -0.028 to -0.004) and current smokers (β = -0.017, 95% CI: -0.027 to -0.007) exhibited notable reductions in WeidnerAge.

Table S5. Subgroup analysis of the association between physical activity and DNA methylation-predicted WeidnerAge.

| Subgroups | β (95% CI) | p-value | p for interaction |
|---|---|---|---|
| Gender | | | 0.663 |
| Male | -0.01(-0.017,-0.002) | 0.013 | |
| Female | -0.006(-0.021,0.008) | 0.377 | |
| Race/ethnicity | | | < 0.001 |
| Non-hispanic White | -0.013(-0.020,-0.005) | 0.002 | |
| Non-hispanic Black | -0.006(-0.015,0.004) | 0.218 | |
| Mexican American | 0(-0.019,0.020) | 0.980 | |
| Other race/ethnicity | 0.012(0.002,0.022) | 0.027 | |
| Marital status | | | 0.207 |
| Never married | 0.011(-Inf,Inf) | 0.268 | |
| Married/living with partner | -0.01(-0.016,-0.004) | 0.002 | |
| Widowed/ divorced | -0.012(-0.037,0.013) | 0.336 | |
| Poverty income ratio | | | 0.051 |
| < 1 | 0.003(-0.004,0.009) | 0.439 | |
| [1,3) | -0.016(-0.028,-0.004) | 0.010 | |
| ≥ 3 | -0.006(-0.014,0.002) | 0.118 | |
| Education | | | 0.798 |
| Below high school | -0.018(-0.044,0.007) | 0.139 | |
| High school | -0.008(-0.018,0.001) | 0.084 | |
| College or above | -0.008(-0.020,0.004) | 0.204 | |
| BMI (kg/m$^2$) | | | 0.52 |
| < 25 | -0.005(-0.022,0.013) | 0.577 | |
| [25, 30) | -0.008(-0.018,0.002) | 0.124 | |
| ≥ 30 | -0.014(-0.024,-0.004) | 0.008 | |
| Smoking status | | | 0.084 |
| Never smoking | 0(-0.010,0.010) | 0.995 | |
| Former smoking | -0.01(-0.022,0.001) | 0.079 | |
| Current smoking | -0.017(-0.027,-0.007) | 0.002 | |
| Alcohol use status | | | 0.857 |
| None | -0.006(-0.019,0.007) | 0.350 | |
| Moderate alcohol use | -0.01(-0.020,0.000) | 0.044 | |
| High alcohol use | -0.008(-0.031,0.015) | 0.459 | |

PA also had a significant effect on VidalBraloAge (Table S6), particularly in males (β = -0.005, 95% CI: -0.009 to 0.000) and non-Hispanic whites (β = -0.007, 95% CI: -0.012 to -0.003). Individuals with a high school education (β = -0.007, 95% CI: -0.012 to -0.001) and a BMI of 30 or greater (β = -0.008, 95% CI: -0.014 to -0.003) also showed significant reductions in predicted age.

Table S6. Subgroup analysis of the association between physical activity and DNA methylation-predicted VidalBraloAge.

| Subgroups | β (95% CI) | p-value | p for interaction |
|---|---|---|---|
| Gender | | | 0.495 |
| Male | -0.005(-0.009,0.000) | 0.039 | |
| Female | -0.007(-0.015,0.000) | 0.051 | |
| Race/ethnicity | | | 0.017 |
| Non-hispanic White | -0.007(-0.012,-0.003) | 0.003 | |
| Non-hispanic Black | 0.001(-0.007,0.009) | 0.734 | |
| Mexican American | -0.002(-0.012,0.008) | 0.719 | |
| Other race/ethnicity | 0.002(-0.004,0.007) | 0.474 | |
| Marital status | | | 0.381 |
| Never married | 0(-Inf,Inf) | 0.885 | |
| Married/living with partner | -0.006(-0.010,-0.002) | 0.002 | |
| Widowed/ divorced | -0.003(-0.015,0.009) | 0.660 | |
| Poverty income ratio | | | 0.224 |
| < 1 | -0.001(-0.008,0.007) | 0.857 | |
| [1,3) | -0.009(-0.017,-0.001) | 0.032 | |
| ≥ 3 | -0.004(-0.008,0.001) | 0.115 | |
| Education | | | 0.119 |
| Below high school | -0.016(-0.034,0.001) | 0.066 | |
| High school | -0.007(-0.012,-0.001) | 0.015 | |
| College or above | -0.001(-0.007,0.006) | 0.850 | |
| BMI (kg/m$^2$) | | | 0.422 |
| < 25 | -0.005(-0.012,0.003) | 0.200 | |
| [25, 30) | -0.003(-0.009,0.003) | 0.383 | |
| ≥ 30 | -0.008(-0.014,-0.003) | 0.005 | |
| Smoking status | | | 0.799 |
| Never smoking | -0.004(-0.009,0.001) | 0.085 | |
| Former smoking | -0.006(-0.012,0.000) | 0.037 | |
| Current smoking | -0.005(-0.011,0.002) | 0.149 | |
| Alcohol use status | | | 0.043 |
| None | -0.008(-0.014,-0.002) | 0.009 | |
| Moderate alcohol use | -0.004(-0.010,0.002) | 0.166 | |
| High alcohol use | 0.005(-0.002,0.011) | 0.147 | |

For ZhangAge (Table S7), PA was inversely associated with predicted age in both males (β = -0.003, 95% CI: -0.006 to -0.001) and females (β = -0.004, 95% CI: -0.006 to -0.001). Non-Hispanic whites (β = -0.005, 95% CI: -0.007 to -0.003) and individuals with a BMI between 25 and 30 (β = -0.005, 95% CI: -0.009 to -0.002) showed strong negative associations with ZhangAge.

Table S7. Subgroup analysis of the association between physical activity and DNA methylation-predicted ZhangAge.

| Subgroups | β (95% CI) | p-value | p for interaction |
|---|---|---|---|
| Gender | | | 0.72 |
| Male | -0.003(-0.006,-0.001) | 0.010 | |
| Female | -0.004(-0.006,-0.001) | 0.004 | |
| Race/ethnicity | | | < 0.001 |
| Non-hispanic White | -0.005(-0.007,-0.003) | <0.001 | |
| Non-hispanic Black | 0.002(-0.001,0.006) | 0.191 | |
| Mexican American | -0.002(-0.005,0.002) | 0.351 | |
| Other race/ethnicity | 0.001(-0.004,0.005) | 0.635 | |
| Marital status | | | 0.033 |
| Never married | 0.002(-Inf,Inf) | 0.337 | |
| Married/living with partner | -0.003(-0.005,-0.001) | 0.004 | |
| Widowed/ divorced | -0.006(-0.011,-0.001) | 0.014 | |
| Poverty income ratio | | | 0.015 |
| < 1 | -0.001(-0.003,0.002) | 0.697 | |
| [1,3) | -0.007(-0.011,-0.003) | <0.001 | |
| ≥ 3 | -0.002(-0.004,0.001) | 0.137 | |
| Education | | | 0.649 |
| Below high school | -0.004(-0.013,0.005) | 0.383 | |
| High school | -0.004(-0.008,-0.001) | 0.019 | |
| College or above | -0.002(-0.006,0.001) | 0.228 | |
| BMI ($kg/m^2$) | | | 0.536 |
| < 25 | -0.002(-0.006,0.002) | 0.261 | |
| [25, 30) | -0.005(-0.009,-0.002) | 0.004 | |
| ≥ 30 | -0.003(-0.008,0.002) | 0.223 | |
| Smoking status | | | 0.715 |
| Never smoking | -0.002(-0.006,0.002) | 0.230 | |
| Former smoking | -0.004(-0.007,0.000) | 0.036 | |
| Current smoking | -0.004(-0.008,-0.001) | 0.010 | |
| Alcohol use status | | | 0.127 |
| None | -0.004(-0.007,-0.001) | 0.024 | |
| Moderate alcohol use | -0.004(-0.007,-0.001) | 0.006 | |
| High alcohol use | 0.002(-0.003,0.007) | 0.412 | |

Finally, for PhenoAge (Table S8), PA was associated with lower predicted ages in both males (β = -0.008, 95% CI: -0.013 to -0.003) and females (β = -0.009, 95% CI: -0.018 to -0.001). Significant reductions were observed in non-Hispanic whites (β = -0.012, 95% CI: -0.017 to -0.006), individuals with a high school education (β = -0.011, 95% CI: -0.018 to -0.004), and current smokers (β = -0.009, 95% CI: -0.016 to -0.001).

Table S8. Subgroup analysis of the association between physical activity and DNA methylation-predicted PhenoAge.

| Subgroups | β (95% CI) | p-value | p for interaction |
|---|---|---|---|
| Gender | | | 0.738 |
| Male | -0.008(-0.013,-0.003) | 0.003 | |
| Female | -0.009(-0.018,-0.001) | 0.032 | |
| Race/ethnicity | | | 0.005 |
| Non-hispanic White | -0.012(-0.017,-0.006) | <0.001 | |
| Non-hispanic Black | 0.007(-0.004,0.018) | 0.199 | |
| Mexican American | -0.002(-0.017,0.014) | 0.833 | |
| Other race/ethnicity | 0.003(-0.011,0.017) | 0.566 | |
| Marital status | | | 0.452 |
| Never married | 0.001(-Inf,Inf) | 0.646 | |
| Married/living with partner | -0.008(-0.014,-0.002) | 0.009 | |
| Widowed/ divorced | -0.008(-0.025,0.008) | 0.313 | |
| Poverty income ratio | | | 0.122 |
| < 1 | -0.001(-0.012,0.009) | 0.766 | |
| [1,3) | -0.014(-0.023,-0.006) | 0.002 | |
| ≥ 3 | -0.004(-0.012,0.003) | 0.248 | |
| Education | | | 0.164 |
| Below high school | -0.01(-0.041,0.021) | 0.502 | |
| High school | -0.011(-0.018,-0.004) | 0.004 | |
| College or above | -0.001(-0.009,0.006) | 0.680 | |
| BMI ($kg/m^2$) | | | 0.812 |
| < 25 | -0.006(-0.015,0.004) | 0.246 | |
| [25, 30) | -0.008(-0.017,0.000) | 0.054 | |
| ≥ 30 | -0.01(-0.020,0.000) | 0.046 | |
| Smoking status | | | 0.988 |
| Never smoking | -0.008(-0.018,0.001) | 0.084 | |
| Former smoking | -0.008(-0.016,0.000) | 0.057 | |
| Current smoking | -0.009(-0.016,-0.001) | 0.026 | |
| Alcohol use status | | | 0.475 |
| None | -0.008(-0.015,-0.002) | 0.015 | |
| Moderate alcohol use | -0.009(-0.016,-0.001) | 0.021 | |
| High alcohol use | 0(-0.015,0.015) | 0.987 | |
